# Mobilization and activation of tumor-infiltrating dendritic cells inhibits lymph node metastasis in intrahepatic cholangiocarcinoma

**DOI:** 10.1038/s41420-024-02079-z

**Published:** 2024-06-26

**Authors:** Bao-Ye Sun, Zhu-Tao Wang, Ke-Zhu Chen, Yang Song, Jing-Fang Wu, Dai Zhang, Guo-Qiang Sun, Jian Zhou, Jia Fan, Bo Hu, Yong Yi, Shuang-Jian Qiu

**Affiliations:** 1 Department of Liver Surgery and Transplantation, Zhongshan Hospital, Fudan University, 180 Fenglin Road, Shanghai, 200032 PR China; 2 The Liver Cancer Institute, Zhongshan Hospital and Shanghai Medical School, Fudan University, Key Laboratory for Carcinogenesis and Cancer Invasion, The Chinese Ministry of Education, 180 Fenglin Road, Shanghai, 200032 PR China; 3 Department of Neurosurgery, Huashan Hospital, Shanghai Medical College, Fudan University, Shanghai, 200040 PR China; 4 Department of Dermatology, Clinical Immunology Research Center, The Second Xiangya Hospital, Central South University, Changsha, 410011 PR China

**Keywords:** Cancer microenvironment, Cancer therapy

## Abstract

Lymph node metastasis (LNM) facilitates distant tumor colonization and leads to the high mortality in patients with intrahepatic cholangiocarcinoma (ICC). However, it remains elusive how ICC cells subvert immune surveillance within the primary tumor immune microenvironment (TIME) and subsequently metastasize to lymph nodes (LNs). In this study, scRNA-seq and bulk RNA-seq analyses identified decreased infiltration of dendritic cells (DCs) into primary tumor sites of ICC with LNM, which was further validated via dual-color immunofluorescence staining of 219 surgically resected ICC samples. Tumor-infiltrating DCs correlated with increased CD8^+^ T cell infiltration and better prognoses in ICC patients. Mechanistically, β-catenin-mediated CXCL12 suppression accounted for the impaired DC recruitment in ICC with LNM. Two mouse ICC cell lines MuCCA1 and mIC-23 cells were established from AKT/NICD or AKT/YAP-induced murine ICCs respectively and were utilized to construct the footpad tumor LNM model. We found that expansion and activation of conventional DCs (cDCs) by combined Flt3L and poly(I:C) (FL-pIC) therapy markedly suppressed the metastasis of mIC-23 cells to popliteal LNs. Moreover, β-catenin inhibition restored the defective DC infiltration into primary tumor sites and reduced the incidence of LNM in ICC. Collectively, our findings identify tumor cell intrinsic β-catenin activation as a key mechanism for subverting DC-mediated anti-tumor immunity in ICC with LNM. FL-pIC therapy or β-catenin inhibitor could merit exploration as a potential regimen for mitigating ICC cell metastasis to LNs and achieving effective tumor immune control.

## Introduction

Intrahepatic cholangiocarcinoma (ICC) accounts for approximately 10–20% of primary liver malignancies, with high mortality and increasing incidence over the past 2 decades globally [1–3]. Surgical resection represents the only potential cure for ICC, while the 5-year survival after surgery remains dismal, mainly attributed to the high incidence of tumor relapse [4, 5]. Among those risk factors, lymph node metastasis (LNM), which was reported to be detected in around 40% of patients undergoing curative-intent resection for ICC, has been positively correlated with rapid tumor recurrence and poor long-term survival [6, 7]. According to the 8th edition of the American Joint Committee on Cancer (AJCC) TNM staging scheme, the presence of LNM indicates more advanced tumor stage in ICC [8]. However, the molecular mechanisms underlying tumor metastasis to lymph nodes remain poorly understood. A thorough characterization of tumor microenvironment could help deepen our understanding of mechanisms associated with LNM and design more effective anti-cancer therapies for ICC.

Tumor immune microenvironment (TIME) is a heterogeneous and dynamically evolving ecosystem composed of lymphocytes and distinct populations of myeloid cells, including monocytes, macrophages, and dendritic cells (DCs) [9, 10]. The TIME plays a critical role in fostering tumor metastasis and determines the treatment response to immunotherapy [11–13], whereas the functional impact of diverse immune cell populations on tumor LNM remain elusive. For effective anti-tumor immunity, conventional DCs (cDCs) fulfill a key role in presenting tumor antigens to sustain T cell-mediated tumor rejection [14, 15]. Tissue-residing cDCs can be divided into two functionally specialized subsets: conventional type 1 DCs (CD103^+^ cDC1s), which especially excel at cross-presenting tumor antigens to prime and activate CD8^+^ T cells, and conventional type 2 DCs (CD11b^+^cDC2s), which are more adept at driving helper CD4^+^ T cell responses [16, 17].The presence of mature DCs (CD83 positive DCs) has been reported to be associated with a significantly lower incidence of LNM and improved survival outcomes in a limited cohort of patients with cholangiocarcinoma [18]. However, it remains uncertain how tumor cells escape cDC-mediated immune surveillance within the TIME and subsequently metastasize to lymph nodes in ICC.

Here, we performed a comparative study of differential immune cell fractions between ICC samples with or without LNM in multiple patient cohorts. We observed decreased infiltration of DCs and CD8^+^ T cells in primary tumor sites of ICC with LNM (LNM-ICC). Tumor-infiltrating DCs and CD8^+^ T cells correlate with better postoperative prognosis in ICC patients. Mechanistically, WNT/β-catenin signaling activation blocks CXCL12 production via β-catenin-mediated transcriptional repression, which results in the defective DC recruitment in LNM-ICC. Moreover, expansion and activation of cDCs at the primary tumor site inhibits LNM in murine ICCs. Our results reveal β-catenin-induced inactivation of CXCL12 expression as a major mechanism underlying impaired DC infiltration into LNM-ICC and provide a rationale for therapeutic strategies aiming at restoring intra-tumoral cDC function to promote effective anti-cancer immunity and abatement of lymph node metastasis.

## Results

### The infiltration of dendritic cells is decreased in primary tumor sites of ICC with LNM

**Fig. 1:**
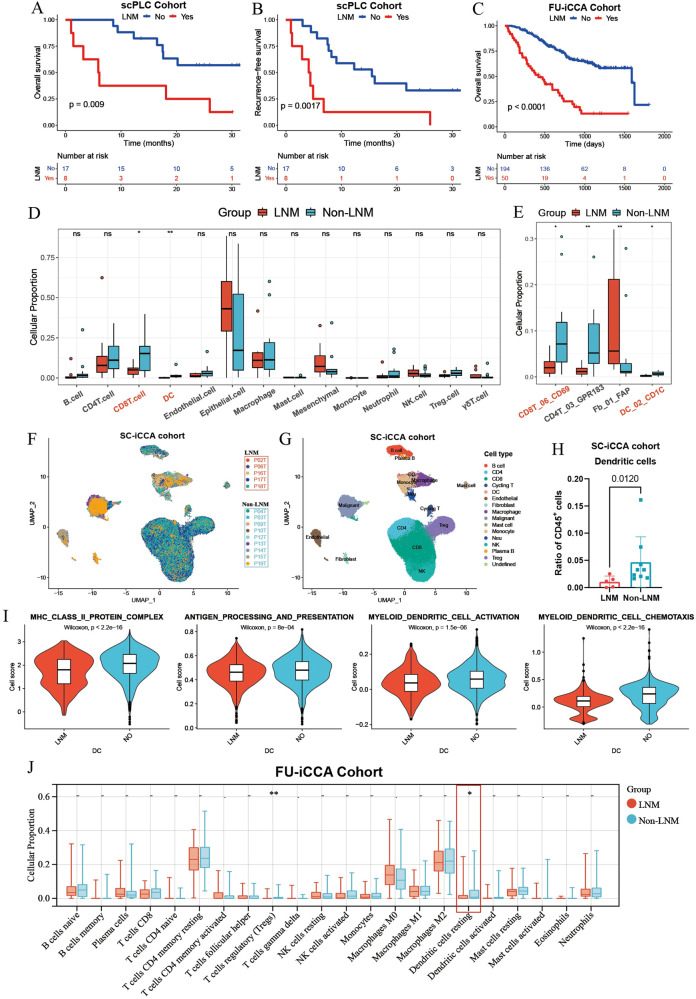
scRNA-seq and bulk RNA-seq analysis of cell type fractions between ICC samples with and without LNM. Kaplan–Meier survival analyses for OS (**A**) and RFS (**B**) of 25 ICC patients with or without LNM in scPLC cohort. **C** Kaplan–Meier survival curves for OS of 244 ICC patients grouped by LNM status in FU-iCCA cohort. *P*-values were determined by log-rank test. Boxplots showing the fractions of 14 major cell types (**D**) and 89 cell clusters (**E**) identified in scRNA-seq analysis of 25 ICC samples with or without LNM. Significance was determined by unpaired Wilcoxon rank-sum test. **F**, **G** UMAP plots visualizing cells from scRNA-seq profiling of 14 ICC samples in SC-iCCA cohort. Samples grouped by LNM status and defined major cell types are distinguished by colors. **H** Boxplot comparing the fractions of dendritic cells between ICC samples with or without LNM in SC-iCCA cohort. **I** The phenotypic scores (MHC class II protein complex, Antigen processing and presentation, Myeloid dendritic cell activation, Myeloid dendritic cell chemotaxis) of dendritic cells between ICC samples with or without LNM in SC-iCCA cohort. Significance was determined by unpaired two-tailed Wilcoxon rank-sum tests. **J** Boxplot comparing the proportions of 22 tumor-infiltrating immune cells estimated by CIBERSORT algorithm using bulk RNA-seq data between ICC samples with or without LNM in FU-iCCA cohort. ICC intrahepatic cholangiocarcinoma, LNM lymph node metastasis, OS overall survival, RFS recurrence-free survival; *p < 0.05, **P < 0.01.

To elucidate the global cellular landscape of ICC tumors with or without LNM, we obtained clinical information of 25 treatment-naïve ICC patients and corresponding statistics of 14 major cell types and 89 cell clusters identified by scRNA-seq analysis of surgically resected ICC samples from scPLC Cohort [19]. As expected, survival analysis revealed that patients with LNM had significantly shorter OS and RFS than those without (Fig. 1A–C). Analysis of 14 major cell type fractions suggested that compared with non-LNM samples, primary ICC tumors with LNM (LNM-ICC) harbored significantly reduced infiltration of CD8^+^ T cells and DCs (Fig. 1D). Further analysis of 89 cell clusters showed that activated CD8^+^ T cell cluster (CD8T_06_CD69) and five DC subtypes, including DC_01_CLEC9A and DC_02_CD1C, were also decreased in LNM-ICC (Fig. 1E and Supplementary Fig. S1A). We then reanalyzed the scRNA-seq data of 14 ICC samples (5 patients with LNM and 9 without) from SC-iCCA cohort [20] (Fig. 1F). 15 major cell types were identified according to highly expressed markers (Fig. 1G and Supplementary Fig. S1B). Consistently, decreased DC fractions were observed in LNM-ICC from SC-iCCA cohort (Fig. 1H). We also observed decreased expression levels of MHC-II antigen-presentation molecules (CD74 and HLA-DP/DQ/DR) in DCs from LNM-ICC (Supplementary Fig. S1C, D). Moreover, DCs in LNM-ICC exhibited significantly decreased phenotypic scores of MHC class II protein complex, antigen processing and presentation, myeloid dendritic cell activation, and myeloid dendritic cell chemotaxis compared with those from non-LN metastatic samples (Fig. 1I), indicating impaired infiltration and function of DCs.

**Fig. 2:**
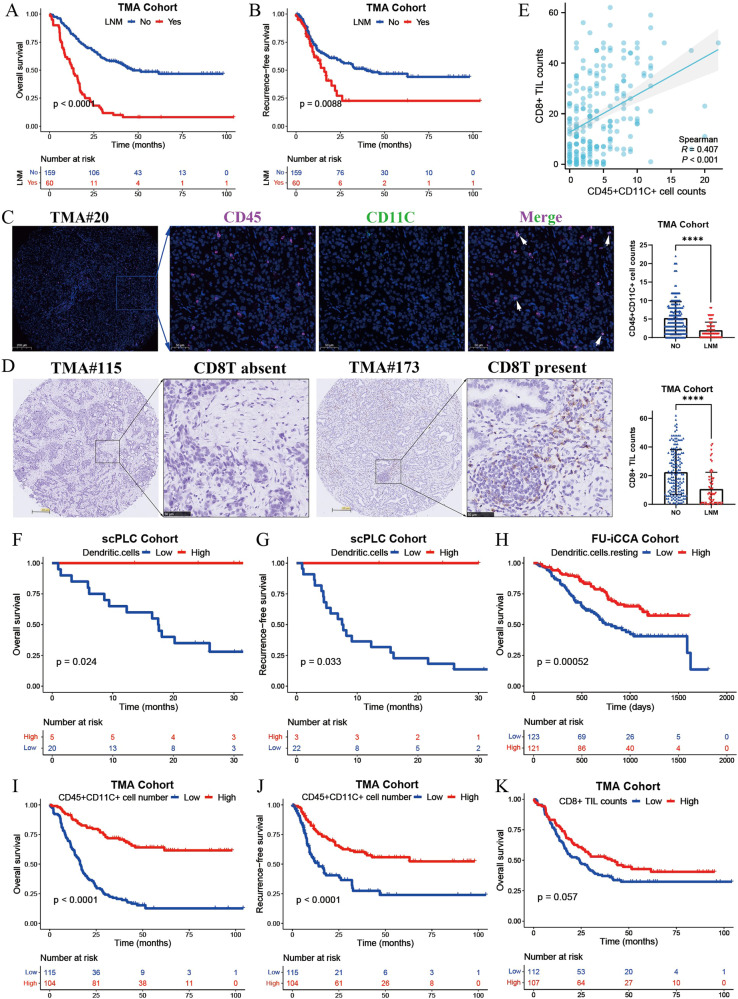
Experimental validation of reduced DCs in ICC samples with LNM and prognostic significance. Kaplan–Meier survival curves for OS (**A**) and RFS (**B**) of 219 ICC patients grouped by LNM status in TMA cohort. **C** Representative immunofluorescence images of CD45^+^ CD11c^+^ DCs (400X, scale bar, 50 μm) and corresponding statistics in surgically resected ICC samples from TMA cohort. White arrows indicate tumor-infiltrating DCs. **D** Representative immunohistochemistry images of positive and negative CD8 staining (400X, scale bar, 50 μm), as well as cell counts of CD8^+^ TILs per field in ICC. **E** Spearman correlation analysis for CD45^+^ CD11c^+^ DCs and CD8^+^ TIL counts in TMA cohort. Kaplan–Meier survival curves for OS (**F**) and RFS (**G**) of 25 ICC patients divided by proportions of intra-tumoral dendritic cells in scPLC cohort. **H** Overall survival analysis of 244 ICC patients grouped by fractions of tumor-resting dendritic cells in FU-iCCA cohort. Kaplan–Meier survival curves for OS (**I**) and RFS (**J**) of 219 ICC patients grouped by cell counts of CD45^+^ CD11c^+^ DCs or CD8^+^ T cells (**K**) in TMA cohort. DCs dendritic cells, TIL tumor-infiltrating lymphocytes, TMA tissue microarray. ****p < 0.0001.

Using CIBERSORT algorithm [21] to deconvolute bulk RNA-seq data from FU-iCCA cohort [22], we calculated the proportion of 22 tumor-infiltrating immune cells in each ICC sample. Similarly, DCs displayed decreased infiltration into LNM-ICC (Fig. 1J). Then we validated these findings using ICC tissue microarrays from TMA cohort. Kaplan–Meier survival analysis showed that patients with LNM had worse clinical outcomes than those without (Fig. 2A, B). Dual-color immunofluorescence (IF) staining and immunohistochemical (IHC) analysis further verified the reduced cell counts of CD45^+^ CD11c^+^ DCs and CD8^+^ T cells in ICC samples with LNM versus those without from the TMA cohort (Fig. 2C, D). A crucial anti-tumor function of DCs involves tumor antigen uptake and presentation to efficiently prime T cell responses. Spearman correlation analysis for expression of CD8^+^ T cell transcript CD8A revealed a positive association with conventional DC marker genes CLEC9A and CLEC10A, along with PD-L1 (CD274) in FU-iCCA cohort (Supplementary Fig. S2A–C). Moreover, the higher DC density detected in ICC specimens also paralleled higher CD8^+^ T cell infiltrates (Fig. 2E), suggesting the critical role of DCs in recruiting and activating tumor-specific CD8^+^ T cells.

Taken together, these results suggested that the defective recruitment of DCs in LNM-ICC could lead to absence of early T-cell priming against tumor-associated antigens and thus promote tumor metastasis to LNs.

### Tumor-infiltrating dendritic cells correlate with better survival outcomes in ICC patients

We next explored the association of tumor-infiltrating DCs with the survival of ICC patients. In scPLC Cohort, higher abundance of DCs indicated significantly better prognoses in patients with ICC, as evidenced by longer OS and RFS (both *P* < 0.05, Fig. 2F, G). In FU-iCCA cohort, patients with higher cellular proportions of tumor-resting dendritic cells had significantly prolonged OS in comparison to those without (Fig. 2H). These results were further validated by IF analysis in TMA cohort (Fig. 2I, J). For CD8^+^ T cells, while no significant differences in OS were observed in FU-iCCA cohort (Supplementary Fig. S2D), the enrichment of intra-tumoral CD8^+^ T cells correlated with better prognosis in ICC patients from TMA cohort (Fig. 2K and Supplementary Fig. S2E). Besides, IHC staining of lymphatic endothelial-specific antibody LYVE-1 suggested that tumor-associated lymphangiogenesis correlated with increased nodal spread and higher recurrence risk (Supplementary Fig. S2F–I), suggesting that approaches targeting lymphangiogenesis could have therapeutic potency in ICC.

### Decreased CXCL12 expression accounts for the defective DC recruitment in LNM-ICC

**Fig. 3:**
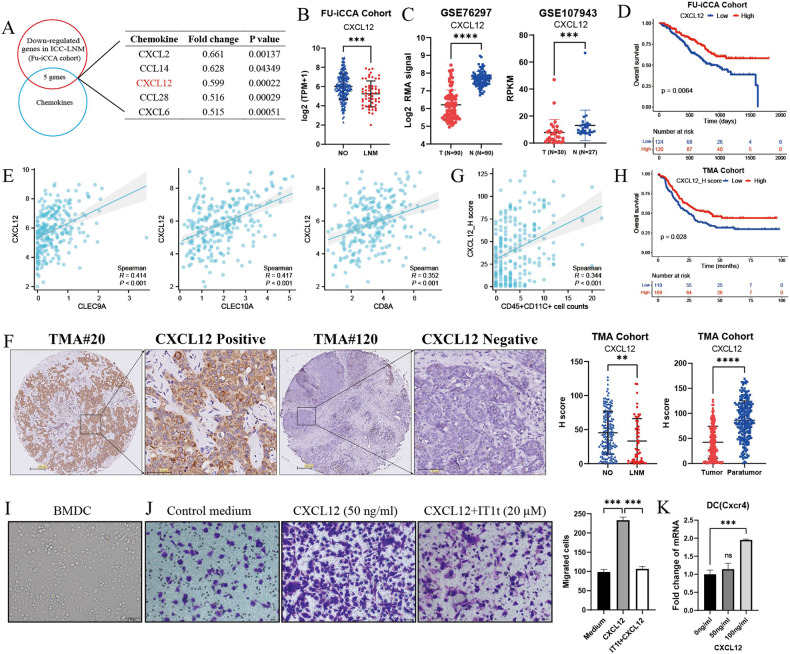
CXCL12 is downregulated in ICC samples with LNM and enhances migration of DCs. **A** Venn diagram displaying the chemokines significantly downregulated in human ICC samples with LNM in FU-iCCA cohort. **B** Scatter plots showing the normalized transcript levels of CXCL12 in ICC samples with or without LNM in FU-iCCA cohort, as well as in adjacent normal liver tissues from datasets GSE107943 and GSE76927 (**C**). **D** Survival analysis of ICC patients grouped by mRNA levels of CXCL12 in FU-iCCA cohort. **E** Spearman correlation analysis of CXCL12 mRNA expression with conventional DC marker genes CLEC9A and CLEC10A, as well as CD8A transcripts in FU-iCCA cohort. **F** Representative IHC images of CXCL12 staining density and quantification by H-score in ICC samples with or without LNM, as well as in adjacent non-tumorous liver tissues from TMA cohort. **G** Spearman correlation of CXCL12 staining density with cell counts of CD45^+^ CD11c^+^ DCs in TMA cohort. **H** Kaplan–Meier survival curves for OS of 219 ICC patients grouped by the median value of CXCL12 H-score in TMA cohort. **I** Bone-marrow-derived dendritic cells (BMDCs) stimulated with recombinant murine GM-CSF (20 ng/ml) and IL-4 (10 ng/ml) for 7 days. **J** Transwell migration assay of BMDCs treated by condition medium and CXCL12 (50 ng/ml), with or without IT1t dihydrochloride (20 µM) for 24 h. **K** qPCR analysis of Cxcr4 in DCs treated by CXCL12 in a concentration-dependent manner. **P < 0.01, ***p < 0.001, ****p < 0.0001.

To pursue mechanisms underlying defective DC recruitment in ICC with LNM, we identified the differentially expressed genes, focusing on expression of chemokines using RNA-seq data from FU-iCCA cohort. Five chemokines (CXCL2, CCL14, CXCL12, CCL28, CXCL6) were significantly downregulated in LNM-ICC samples (Fig. 3A, B). Differentially expressed gene analysis of single malignant cells in SC-iCCA cohort also revealed reduced CXCL12 gene expression in LNM-ICC, as well as CCL4 and CCL5 (Supplementary Fig. S3A–C), which were reported to be DC chemoattractants [23, 24]. Since CXCL12, also known as stromal-derived factor-1 (SDF-1), has been reported to affect the migration of DCs [25, 26], we further investigated the association between CXCL12 expression and impaired DC recruitment in LNM-ICC. Reduced levels of CXCL12 transcripts were also observed in ICC compared with normal liver tissues in two independent GEO datasets (Fig. 3C). Survival analysis revealed that high expression of CXCL12 mRNA was associated with better clinical outcomes in FU-iCCA cohort (Fig. 3D). Moreover, CXCL12 expression positively correlated with conventional DC transcripts CLEC9A and CLEC10A, as well as CD8A (Fig. 3E). We further confirmed the decreased CXCL12 protein levels in LNM-ICC samples compared with non-LNM samples by IHC staining in TMA cohort (Fig. 3F). Meanwhile, CXCL12 protein levels were downregulated in ICC tumors compared with paired adjacent non-tumorous liver tissues (Fig. 3F). Additionally, a positive correlation between CXCL12 staining density and CD45^+^CD11C^+^ DC or CD8^+^ T cell counts (Fig. 3G and Supplementary Fig. S3D), along with a significantly better OS in patients with high expression of CXCL12 was observed (Fig. 3H).

To validate the functional role of CXCL12, we performed an in vitro trans-well migration assay of mouse bone-marrow-derived dendritic cells (BMDCs) in response to recombinant murine CXCL12. The migration of BMDCs was enhanced by CXCL12 treatment, which was abrogated by antagonist of its corresponding chemokine receptor CXCR4 (Fig. 3I, J). Besides, CXCL12 increased CXCR4 expression in a concentration-dependent manner (Fig. 3K). Together, our findings indicate that the reduced recruitment of DCs into the primary tumor sites of LNM-ICC could be partially explained by a defective production of the chemokine CXCL12.

### β-catenin activation impairs DC recruitment via transcriptional repression of CXCL12 in LNM-ICC

**Fig. 4:**
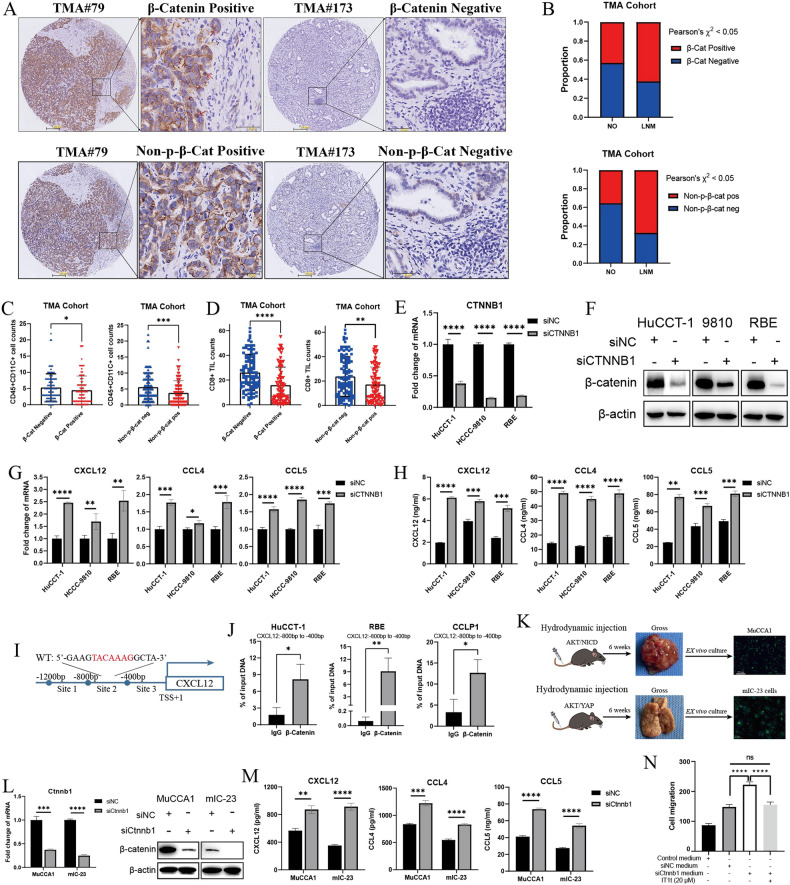
β-catenin signaling activation excludes DC infiltration by suppressing CXCL12 expression in ICC with LNM. **A** Representative IHC staining images of β-catenin or non-phosphorylated β-catenin in ICC samples from TMA cohort. Red arrows indicate positive nuclear staining of β-catenin. **B** Association between β-catenin or non-phosphorylated β-catenin staining and LNM status in TMA cohort. Cell counts of CD45^+^ CD11c^+^ DCs (**C**) and CD8^+^ TILs (**D**) in ICC samples grouped by β-catenin or non-p-β-catenin staining intensity in TMA cohort. The mRNA (**E**) and protein expression (**F**) of CTNNB1 (β-catenin) assessed by qPCR and western blot in three human ICC cell lines 48 h following siRNA transfection. CXCL12, CCL4, and CCL5 mRNA expression (**G**) quantitated by qRT-PCR and amount of secreted CXCL12, CCL4, and CCL5 (**H**) in 48h-conditioned siRNA-treated human ICC cell supernatants detected by enzyme-linked immunosorbent assay (ELISA). **I** Putative β-Catenin/LEF1/TCF complex binding site (5’-TACAAAG-3’) in the promoter region (site 2) of CXCL12 was identified by in silico analysis. Specific primers for this promoter region (−800bp to −400bp) were designed. **J** ChIP-qPCR assay was performed in ICC cell lines with IgG and β-Catenin-specific antibodies. The results are presented as percentage of the total input chromatin DNA. **K** Schematic representation for the establishment of mouse ICC cell lines MuCCA1 and mIC-23 cells. MuCCA1 cells were generated from AKT/NICD-driven ICCs after 6 months of ex vivo culture. mIC-23 cells were generated from AKT/YAP-induced ICCs. Anti-CK19 antibody was used for IF staining of ICC cells. **L** Knockdown efficiency validated by qRT-PCR and western blot in two mouse ICC cell lines 48 h following siRNA transfection. **M** Secreted CXCL12, CCL4, and CCL5 in 48h-conditioned siRNA-treated mouse ICC cell supernatants detected by ELISA. **N** Trans-well migration assay of BMDCs attracted by condition medium and siRNA-treated MuCCA1 cell supernatants, with or without IT1t dihydrochloride (20 µM) for 24 h. *p < 0.05, **P < 0.01, ***p < 0.001, ****p < 0.0001.

We next sought to identify the mechanism underlying suppressed CXCL12 expression, which could result in the defective DC recruitment in LNM-ICC. Gene Set Enrichment Analysis (GSEA) of RNA-seq data from FU-iCCA cohort showed that Hallmark gene sets like MTORC1 signaling, Glycolysis, Hypoxia, WNT/β-catenin signaling, and Reactome pathway TCF-dependent signaling in response to WNT were enriched in ICC samples with LNM (Supplementary Fig. S4A, B). Previous reports had suggested that β-catenin activation suppresses the intra-tumoral recruitment of DCs by inhibiting chemokines CCL4 and CCL5 production [23, 24]. Moreover, CXCL12 and CCL5 were downregulated in human CTNNB1(β-catenin)-mutant HCC samples (Supplementary Fig. S4C, D). Thus, we continued to investigate whether decreased DC infiltration was associated with active β-catenin signaling in LNM-ICC. IHC staining of both β-catenin and non-phosphorylated β-catenin (activated form of β-catenin) in human ICC samples from TMA cohort suggested that β-catenin activation correlated with LNM status in ICC patients (Fig. 4A, B). Furthermore, active β-catenin signaling was significantly associated with reduced infiltration of CD45^+^ CD11c^+^ DCs and CD8^+^ T cells in ICC (Fig. 4C, D). Short interfering RNA (siRNA)-mediated knockdown of β-catenin in human ICC cell lines restored CXCL12 production, with concomitant CCL4 and CCL5 upregulation (Fig. 4E–H).

Activation of β-catenin caused its stabilization and subsequent translocation to the nucleus, where it forms a complex with transcription factors LEF/TCF that regulate expression of several target genes [27]. Our results also suggested that β-catenin suppresses CXCL12 expression at the transcriptional level (Fig. 4G). To investigate the mechanism by which β-catenin signaling inactivates CXCL12 transcription, we performed in silico analysis of CXCL12 gene promoter sequences. This identified a putative LEF1/TCF binding motif (5’-TACAAAG-3’) [28] in the human CXCL12 gene promoter located between positions −800 to −400 bp relative to the transcription start site (Fig. 4I). Specific primers for this promoter region were designed. A chromatin immunoprecipitation (ChIP) assay further confirmed the binding of β-catenin to the CXCL12 promoter region in ICC cell lines (Fig. 4J). To study the impact of tumor cell intrinsic β-catenin signaling on mouse BMDC chemotaxis, we generated two mouse ICC cell lines MuCCA1 and mIC-23 cells from AKT/NICD or AKT/YAP-driven murine spontaneous ICCs respectively (Fig. 4K). Hydrodynamic tail vein injection (HDTVi) of indicated plasmids was used to generate the AKT/NICD or AKT/YAP-induced mouse ICC models. H&E and IHC analysis confirmed the spontaneous induction of ICC, including positive staining of biliary-specific marker cytokeratin (CK)-19 and negative staining of hepatocytic-specific transcription factor HNF4α (Fig. S4E and S4H). Cellular IF staining showed that MuCCA1 and mIC-23 cells universally expressed CK19 (Fig. S4F and S4I). Moreover, MuCCA1 and mIC-23 subcutaneous tumors from C57BL/6 mice displayed intense desmoplastic reaction and typical immunohistopathological features of cholangiocarcinoma, as evidenced by CK19 and a-SMA positivity and HNF4α negativity (Fig. S4G and S4J). Consistently, siRNA-induced silencing of β-catenin in two mouse ICC cell lines promoted the secretion of chemokines CXCL12, CCL4 and CCL5 (Fig. 4L-M). Trans-well migration assays revealed that cell supernatants from MuCCA1 and mIC-23 cells undergoing knockdown of β-catenin promoted the migration of BMDCs, whereas the addition of CXCR4 antagonist abolished the migration activity (Fig. 4N and S4K).

As WNT and CTNNB1 mutations are rare in ICC patients [29], it is most likely that β-catenin signaling is activated via the WNT ligands from the tumor microenvironment. Indeed, we observed increased expression of multiple WNT ligands, FZD receptors, and CTNNB1 in human ICC samples compared with nontumorous surrounding liver tissues based on the RNA-seq dataset GSE107943 (Fig. S4L and S4M). Our data supported the activation of β-catenin in human ICC, which is likely WNT-ligand dependent. Previous studies showed that cancer-associated fibroblasts (CAFs) might be the major source of WNT ligands [30], and CAFs promote tumor lymphangiogenesis in cholangiocarcinoma and immune evasion [31–33]. We have also reported that hepatic stellate cells (HSCs) promote ICC progression via WNT/β-catenin signaling axis [34]. More importantly, FAP^+^ CAF subset (Fb_01_FAP) displayed significantly higher infiltration into primary ICC tumors with LNM (LNM-ICC) compared with non-LNM samples in scPLC Cohort (Fig. 1E). Spearman correlation analysis also revealed the negative association between Fb_01_FAP and the infiltration of DC and CD8 T cells (Fig. S4N). These findings suggested that FAP^+^ CAFs could play a vital role in fostering tumor LNM and escaping dendritic cell-mediated anti-tumor immunity in ICC.

Collectively, our results suggest that WNT/β-catenin signaling activation within LNM-ICC blocks CXCL12 production, which is partly mediated through β-catenin-dependent transcriptional repression.

### Combined Flt3L and poly I:C therapy inhibits lymph node metastasis of mIC-23 footpad tumor

**Fig. 5:**
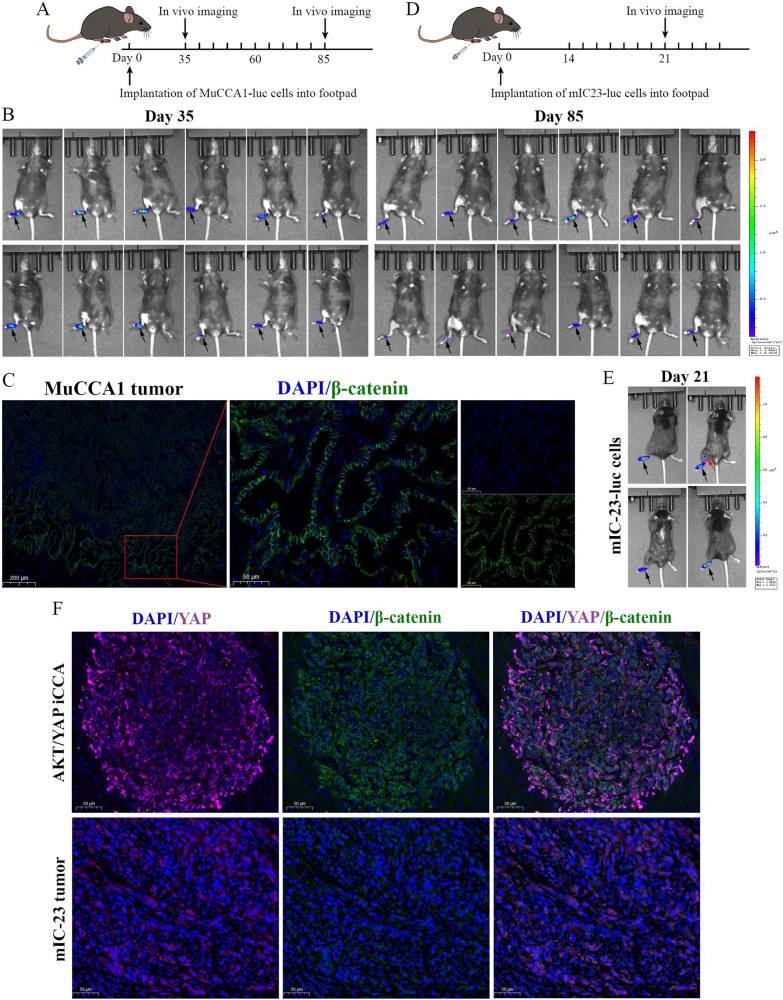
Exploration of mouse ICC cell lines used to construct the footpad tumor LNM model. **A** Schematic representation for the establishment of MuCCA1 footpad tumor model. **B** In vivo bioluminescence imaging was used to monitor primary footpad tumor and popliteal LN metastasis on Day 35 and Day 85. **C** Immunofluorescence staining of DAPI and β-catenin in MuCCA1 footpad tumor tissues. **D** Schematic representation for the establishment of mIC-23 footpad tumor model. **E** In vivo imaging used to monitor primary footpad tumor (black arrow) and popliteal LN metastasis (red arrow) on Day 21. **F** Immunofluorescence staining of DAPI, YAP, and β-catenin in AKT/YAP-induced iCCA sections and mIC-23 tumor tissues.

Systemic injection of Fms-like tyrosine kinase 3 ligand (Flt3L) has been reported to induce the expansion of immature cDCs at the tumor site [15], whereas immature DCs can’t efficiently prime T cells. Therefore, we combined the administration of a toll-like receptor 3 (TLR3) agonist poly(I:C) to promote the maturation and activation of Flt3L-mobilized DCs [15, 35, 36]. We first explored whether MuCCA1 cells could be used for the footpad tumor LNM model (Fig. 5A). However, dynamic in vivo imaging on Day 35 and Day 85 revealed that MuCCA1 cells showed limited LNM potential and therefore was not suitable to construct the LNM model (Fig. 5B), possibly due to the lack of β-catenin signaling activation as evidenced by membrane β-catenin IF staining (Fig. 5C). Then, we turned to another mouse ICC cell line mIC-23 established from AKT/YAP-induced ICC and found that mIC-23 cells could be used to construct the tumor LNM model (Fig. 5D-E). Furthermore, IF staining of YAP and β-catenin in AKT/YAP-driven ICC lesions and mIC-23 footpad tumors revealed the simultaneous nuclear colocalization of YAP and β-catenin (Fig. 5F), suggesting the full activation of both YAP and β-catenin signaling.

**Fig. 6:**
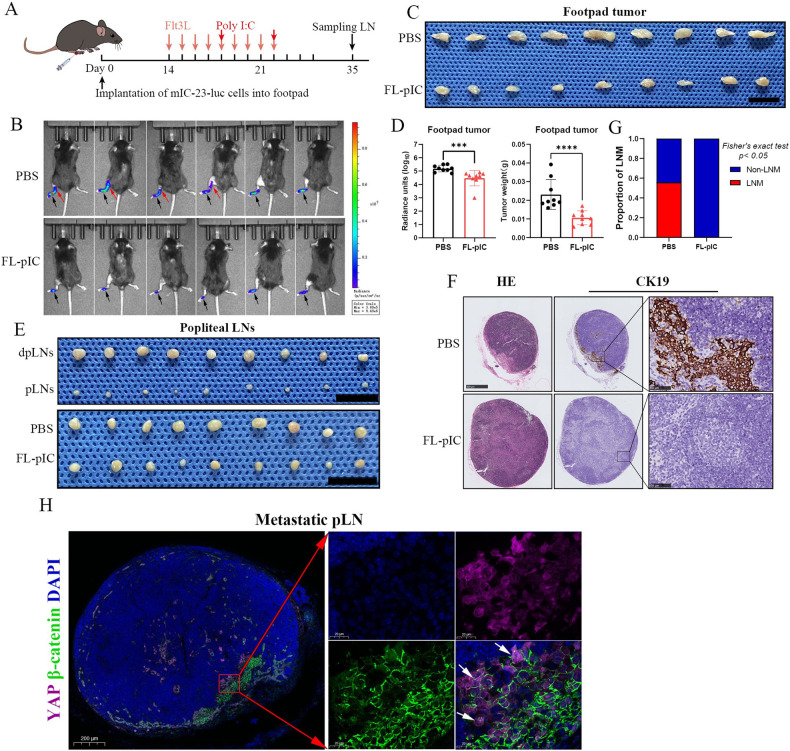
Combined Flt3L and poly I:C therapy inhibits lymph node metastasis of mIC-23 footpad tumor. **A** Treatment schedule for Flt3L combined with high molecular weight poly(I:C) in mIC-23 footpad implantation model. **B** In vivo bioluminescence imaging was used to monitor primary footpad tumor (black arrow) and popliteal LN metastasis (red arrow). **C** Gross images of footpad tumors from indicated groups. Scale bar: 1 cm. **D** Quantification of normalized luciferase signal (left) and weight (right) of footpad tumors. **E** Gross images of popliteal LNs from indicated groups. H&E staining and IHC staining for CK19 of popliteal LNs (**F**) and incidence of LN metastasis (**G**) in indicated groups. **H** Immunofluorescence staining of DAPI, YAP, and β-catenin in metastatic popliteal LNs. White arrows indicate nuclear co-localization of YAP and β-catenin in metastatic ICC cells.

We next investigated the therapeutic effects of combined FL-pIC therapy on LNM in vivo using the mIC-23 footpad tumor LNM model. Mice were randomly divided into 2 groups (*n* = 9) and received indicated treatment after implantation of luciferase (Luc)-labeled mIC-23 cells into the footpad region (Fig. 6A). In vivo bioluminescence imaging revealed that compared with PBS group, FL-pIC therapy significantly suppressed the metastasis of mIC-23 tumor cells to popliteal LNs (Fig. 6B). The primary footpad tumors, popliteal LNs and inguinal LNs were harvested. Consistently, FL-pIC treatment inhibited the growth of mIC-23 footpad tumors, as revealed by the obvious reduction in luciferase signals and tumor weight in FL-pIC group (Fig. 6C, D). We also observed a larger volume of tumor-draining popliteal LNs than contralateral normal LNs (Fig. 6E). H&E staining and IHC staining for CK19 of popliteal LNs further validated that FL-pIC therapy markedly suppressed LN metastasis without affecting the size of the tumor-draining popliteal LNs (Fig. 6E–G). Besides, IF staining of popliteal LNs exhibited a positive nuclear staining of YAP and β-catenin in metastatic mIC-23 cells (Fig. 6H).

### β-catenin inhibition restored DC infiltration and suppressed LN metastasis of mIC-23 cells

**Fig. 7:**
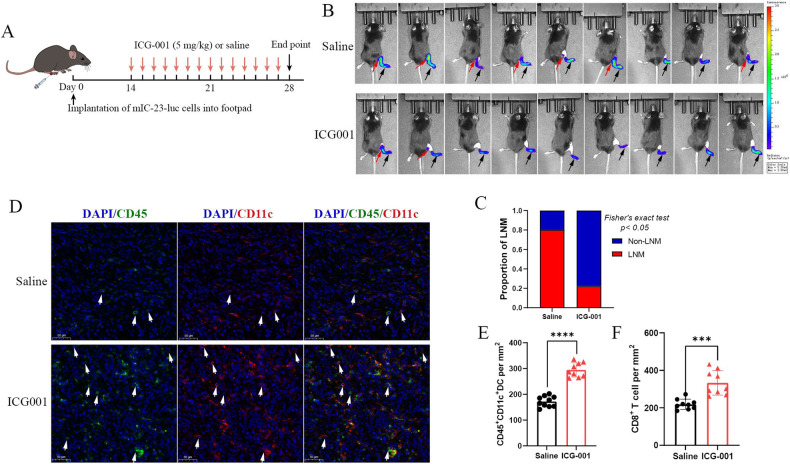
β-catenin inhibitor ICG-001 promoted intra-tumoral DC infiltration and suppressed LN metastasis of mIC-23 cells. **A** Treatment schedule for β-catenin inhibitor ICG-001 in mIC-23 footpad tumor model. **B** In vivo bioluminescence imaging was used to monitor primary footpad tumor (black arrow) and popliteal LN metastasis (red arrow). **C** Proportion of popliteal LN metastasis from indicated groups. Representative immunofluorescence staining images of CD45^+^ CD11c^+^ DCs (**D**) and corresponding statistics (**E**) in mIC-23 footpad tumors from indicated groups. **F** Quantification of CD8^+^ T cells in mIC-23 footpad tumors from indicated groups.

At last, we explored the impact of β-catenin inhibition on intra-tumoral infiltration of DCs and LNM using the mIC-23 footpad tumor model (Fig. 7A). We found that β-catenin inhibitor ICG-001 effectively suppressed the popliteal LN metastasis of mIC-23 cells (Fig. 7B, C). Concomitantly, β-catenin inhibition rescued the defective DC and CD8^+^ T cell infiltration into primary tumor sites, as indicated by the dual-color IF staining and IHC analysis (Fig. 7D–F and Supplementary Fig. S5).

## Discussion

For many solid cancers, metastasis of tumor cells to lymph nodes often predicts subsequent dissemination to distant organs, leading to the high mortality of cancer patients [37]. Previous studies have focused on the mechanism by which tumor cells spread and adapt to the lymph node microenvironment, such as metabolic shift towards fatty acid oxidation [38] and MHC-I and PD-L1 upregulation [39]. Besides malignant cells, the functional impact of primary TIME on tumor LN metastasis remains poorly understood. In the present study, we have demonstrated β-catenin-induced CXCL12 suppression as an important mechanism explaining the defective DC recruitment in ICC samples with LNM. Mobilization and activation of cDCs by combined FL-pIC therapy or inhibition of β-catenin signaling remarkably suppresses lymph node metastasis in murine ICCs.

As the most potent antigen presenting cells (APCs) for T-cell priming, conventional DCs play a crucial role in orchestrating effective anti-tumor immunity [14, 15]. However, DCs are sparsely distributed, rarely infiltrated into the tumor lesions, and are often programmed in a dysfunctional state by immunosuppressive factors such as tumor-derived prostaglandin E2 (PGE2) and granulocyte colony-stimulating factor (GCSF) [15, 40, 41]. The paucity of tumor-infiltrating DCs leads to dysfunctional immune surveillance in cancers [36, 41]. In our study, infiltration of DCs was significantly decreased in ICC with LNM. Besides, cDCs are associated with increased intra-tumoral CD8^+^ T cell infiltration and improved patient survival. Moreover, scRNA-seq analysis suggested that the classical phenotypic functions of DCs, such as antigen processing and presentation, myeloid dendritic cell activation and chemotaxis, were impaired in DCs in LNM-ICC samples. This defective recruitment and function of tumor-infiltrating DCs could in turn impaired the presence of antigen-specific CD8^+^ T cells, which confers tumor cells enhanced LN metastatic potential and resistance to anti-PD-1 therapies. Therefore, strategies aiming to increase the abundance and restore the function of tumoral cDCs hold great potential to elicit effective anti-cancer T cell responses.

Systemic administration of Flt3L, followed by a TLR3 agonist poly(I:C) or CD40 agonist, has been reported to induce the expansion and activation of tumor-residing cDC1s and enhances tumor responses to anti-PD-L1 therapy [15, 35]. In situ vaccination (ISV) combining Flt3L, local radiotherapy, and a TLR3 agonist, led to systemic clinical cancer remission and potentiation of PD-1 blockade in patients with advanced stage indolent non-Hodgkin’s lymphomas (iNHLs) [42]. CD40 agonist-mediated activation of macrophages and dendritic cells also enhances response to anti-PD-1 therapy in murine ICCs [43]. Using an AKT/YAP-induced spontaneous ICC model [43, 44], we established a mouse ICC cell line mIC-23, which displayed typical immunohistopathological features of ICC. Moreover, mIC-23 cells could be used to construct the footpad tumor LNM model. We thus tested the effects of combined FL-pIC therapy on the LNM potential of mIC-23 cells in vivo and found that FL-pIC therapy effectively inhibited the metastasis of mIC-23 cells to LNs. This indicated that restoring function of tumor-infiltrating DCs could be one option to suppress LN metastasis and subsequent distant metastasis. Our findings substantiate that recruiting and activating intra-tumoral DCs is feasible and critical for effective immune surveillance.

Tumor-intrinsic activation of WNT/β-catenin pathway can mediate cancer immune evasion. β-catenin activation results in T-cell exclusion and resistance to anti-PD-1 therapy in multiple cancers [23, 24]. β-catenin activation can also suppress the recruitment of tumor-infiltrated dendritic cells and activated B cells [23, 24, 45]. As CTNNB1 mutations are extremely rare in ICC patients [29], we postulated that β-catenin signaling is activated via other pathways, for example, the WNT ligands from the tumor microenvironment. The discovery of β-catenin activation in human ICC samples is the major conclusion of a previous study from professor Xin Chen, which claimed that non-mutant form of β-catenin is indispensable for YAP-driven cholangiocarcinoma formation [30]. To further investigate this important question, we evaluated the activation status of β-catenin using IHC staining of non-phosphorylated β-catenin in human ICC specimens from TMA cohort. Our findings corroborated that β-catenin is activated in ICC and correlates with reduced infiltration of CD8^+^ T cells and DCs.

Several studies have also revealed the negative association between chemokine expression and canonical WNT/β-catenin signaling. In melanoma, colorectal cancer, and non-small cell lung carcinoma (NSCLC), the reduced recruitment of intra-tumoral CD103^+^ DCs was caused by β-catenin-mediated inhibition of chemokine CCL4 production [23, 46, 47]. In the context of HCC, β-catenin activation impairs DC recruitment through suppressing CCL5 expression [24, 48]. Previous studies have revealed the down-regulation of CXCL12, a chemoattractant for DCs [25, 26], induced by canonical WNT/β-catenin signaling [45, 49]. Consistently, we observed that β-catenin signaling blocked expression of CXCL12, CCL4 and CCL5 in ICC cells, which could account for the defective DC infiltration in LNM-ICC.

In conclusion, our findings suggest that β-catenin activation results in DC exclusion into ICC tumor microenvironment with LNM by blocking CXCL12 production. Expansion and activation of intra-tumoral cDCs represents a promising therapeutic strategy for mitigating tumor cell metastasis to LNs.

## Materials and methods

### Patient cohorts enrolled

This study included four ICC patient cohorts. (1) scPLC Cohort [19] included scRNA-seq data of 160 samples from 124 treatment-naive patients with primary liver cancer (PLC), including 79 with hepatocellular carcinoma (HCC), 25 with ICC and 7 with CHC. We obtained clinical information and statistics of 14 major cell types and 89 cell clusters in scRNA-seq samples of 25 ICC patients. (2) For SC-iCCA Cohort [20], we reanalyzed scRNA-seq data of 14 ICC samples, including 5 samples with LNM and 9 without LNM. (3) The Bulk RNA-seq data of 255 ICC samples from Zhongshan Hospital, Fudan University (FU-iCCA cohort [22]) were analyzed as previously described [50]. (4) A tissue microarray composed of ICC specimens (TMA cohort) from Zhongshan Hospital was constructed. The TMA cohort recruited 219 patients with pathologically diagnosed ICC who received curative liver resection between 2012 and 2017. All studies involving human specimens were conducted in accordance with the institutional guidelines of the Research Ethics Committee of Zhongshan Hospital. Written informed consent was obtained from each patient for use of tissue samples and clinical information.

### Animals

All animal studies were performed in compliance with the experimental protocols approved by the Institutional Animal Care and Use Committee of Zhongshan Hospital, Fudan University. Six-to eight-week-old C57BL/6 mice were purchased from Shanghai Ji-hui Experimental Animal Breeding Co., Ltd. All mice were housed under specific pathogen-free conditions in the animal center of Fudan University.

### AKT/NICD and AKT/YAP-induced mouse ICC model

Hydrodynamic tail vein injection (HDTVi) of indicated plasmids was used to generate the murine ICC models [43, 44]. To establish AKT/NICD-induced spontaneous ICCs, 20 μg pT3-EF1a-NICD (Addgene plasmid #46047), 20 μg PT3-myr-AKT-HA (Addgene plasmid #31789) and 10 μg pCMV-CAT-T7-SB100 (Addgene plasmid #34879) per mouse dissolved in 2 mL Ringer solution were injected into C57BL/6 mice through tail-vein injection within 5–7 s. Plasmids were amplified in E. coli cultures and purified using Plasmid MaxiPrep (endotoxin-free) Kits. For AKT/YAP-induced spontaneous murine ICCs, 8-week-old C57BL/6 mice underwent hydrodynamic tail vein injections with 20 μg of AKT plasmid or AKT fused with luciferase gene fragment (AKT-luc), 30 μg of YAP (pT3-EF1a-FLAG-YAPS127A; Addgene plasmid #46049) and 10 μg of SB100 dissolved in 2 mL Ringer solution.

### Tumor induction and establishment of two mouse ICC cell lines

For AKT/NICD or AKT/YAP-induced murine ICCs, liver tumors were harvested six weeks after plasmid injection. Fresh mouse ICC tissues were cut into small pieces and enzymatically digested with 0.5 mg/ml collagenase I and IV (Gibco) for 30 min. The tissues were then dissociated into single cells using the gentleMACS Dissociator (Miltenyi Biotec). Dissociated tumor material was filtered through a sterile 70-μM cell strainer and plated in a T25 cell culture flask containing high-glucose DMEM supplemented with 10% FBS. Attached cells were trypsinized and re-plated when near-confluence every 3~4 days. After 6 months of ex vivo culture and serial passage, two murine ICC cell lines, MuCCA1 (derived from AKT/NICD-induced ICC) and mIC-23 (derived from AKT/YAP-induced ICC), were established and used for further functional experiments.

### Subcutaneous tumor injection model

Six-week-old female C57BL/6 mice were injected subcutaneously in the left flank with 5 ×10^6^ MuCCA1 or mIC-23 cells suspended in 100 μL PBS. On the 35th day after cell implantation, mice were sacrificed and tumors were harvested for further analyses.

### mIC-23 cell footpad implantation model

For mIC-23 cell footpad implantation model. 1 ×10^6^ luciferase (Luc)-labeled mIC-23 cells in 20 μL PBS were implanted subcutaneously into the footpad region of hind limb of 6-week-old female C57BL/6 mice. The treatment was started from the second week after tumor cell implantation. Primary tumor volume and tumor LN metastasis were monitored by in vivo bioluminescence imaging. At the experimental endpoint, primary tumor tissues and ipsilateral popliteal LNs (pLNs) were sampled and analyzed for tumor metastasis by Hematoxylin-eosin (HE) staining and immunohistochemistry.

### Treatments

For expansion and activation of DCs, we applied combined Flt3L and poly(I:C) therapy. C57BL/6 tumor-bearing mice were treated intraperitoneally with 30 μg active recombinant mouse Flt3L Protein (RP01058, ABclonal Technology) dissolved in 100 μL PBS or control PBS for 9 consecutive days as previously published [15, 35]. High molecular weight poly(I:C) (InvivoGen) was injected intratumorally (50 μg/dose in 30 μl PBS) on day 5 and 9 after Flt3L administration [15]. For β-catenin inhibition, mice were treated intraperitoneally with ICG-001 (5 mg/kg) or saline. Mice were monitored routinely to examine tumor formation and treatment tolerability. At the endpoint of the indicated treatment, mice were sacrificed and tumor tissues were harvested for further analyses.

### In vivo imaging

To monitor tumor formation and LN metastasis, we performed in vivo bioluminescence imaging. Mice were anesthetized and received intraperitoneal injection of 150 mg/kg D-luciferin (D115509, aladdin). After 10 min, in vivo imaging was performed using an IVIS Spectrum system (Perkin Elmer). Luciferase signal was quantified as average Radiance [Photons/second/cm^2^/sr] of region of interest using Living Image software 4.4 (Perkin Elmer).

### Cell lines

Human ICC cell lines RBE, HCCC-9810, HuCCT-1, and CCLP1 were obtained from the Liver Cancer Institute, Fudan University. Mouse ICC cell lines mIC-23 and MuCCA1 cells were established in our laboratory as described above. RBE, HCCC-9810, HuCCT-1 cells were cultured in RPMI 1640 medium supplemented with 10% FBS (Gibco) in a 37 °C humidified incubator with 5% CO2. CCLP1, mIC-23 and MuCCA1 cells were cultured in DMEM containing 10% FBS.

### RT-qPCR

Total RNA was extracted using TRIzol (Invitrogen) and reverse transcription was performed using PrimeScript RT Master Mix (Takara, Japan, RR036A). qPCR was performed using a TB-Green-based PCR kit (Takara, Japan) on a Real-Time PCR system (Applied Biosystems, USA). β-actin was used as an internal control. The primers are listed in Supplementary Table S1. The relative quantification (2^−ΔΔCt^) method was applied to analyze the fold-change in expression levels relative to a control sample.

### Chromatin immunoprecipitation (ChIP) assay

For ChIP assays, cells were grown to 90% confluence in a 10 cm Petri dish. Cells were fixed with 1% formaldehyde solution for 10 min at room temperature. Subsequent steps were performed using the SimpleChIP® Enzymatic Chromatin IP Kit (CST, Cat#9003S) following the manufacturer’s instructions. Chromatin-containing supernatants were incubated with β-catenin antibody (1:25 dilution, Cat# 8480S, CST) or normal rabbit IgG overnight. The immunoprecipitated DNA and total input chromatin DNA (1:10 dilution) was then used as template for real-time qPCR using indicated primers. The primers specific for the promoter region of human CXCL12 gene used in the ChIP assay are listed in Supplementary Table S2. The amount of pulled-down DNA in each sample is presented as percentage of the total amount of input chromatin DNA, which were calculated as followed: ΔCT[ChIP] = Ct [ChIP] − (Ct [Input] − Log_2_[Input dilution factor = 10]), percentage of Input DNA = 2^−ΔCT^*100.

### RNAi and transfection

For short interfering RNA (siRNA)-mediated knockdown of CTNNB1, human CTNNB1 siRNA (si-CTNNB1), mouse siCtnnb1, and non-targeting siRNA as negative control (si-NC) vectors were synthesized by Tsingke Biotech (Beijing, China). Sequences that specifically target human CTNNB1 and mouse Ctnnb1 are listed in Supplementary Table S3. For siRNA transfection, 1 ×10^6^ tumor cells were cultured in 6-well plates. 250 μL of Opti-MEM (Gibco) was mixed with 25 pmol siRNA and 7.5 μL Lipofectamine® RNAiMAX reagent (Invitrogen) per well and added to the cell culture. Cells were incubated for 48 h before supernatant was harvested for ELISA assays and cells were collected for RNA or protein extraction.

### Generation of bone-marrow-derived dendritic cells (BMDCs)

For generation of BMDCs, bone marrow from C57BL/6 female mice was collected from the femurs and tibias of both legs. After washing and lysis of erythrocytes, bone marrow cells were cultured in RPMI 1640 medium containing 10% FBS incubated with recombinant murine GM-CSF (PeproTech, 20 ng/ml) and IL-4 (PeproTech, 10 ng/ml) for 7 days.

### Trans-well migration assays

For migration assays, 1 ×10^5^ BMDCs were seeded in the upper chamber (24-well insert, Corning, USA) with murine SDF-1beta /CXCL12 (PeproTech, 50 ng/ml) and/or CXCR4 antagonist IT1t dihydrochloride (MCE, 20 µM). After culturing for 24 h, cells from the lower compartment were fixed with 4% paraformaldehyde and stained with crystal violet. Photographs were then taken under 5 independent microscopic fields (200X) and stained cells were counted.

### ELISA

ELISA assays against human and mouse CCL4, CCL5, and CXCL12 in cell supernatant were performed using specific ELISA kits (NEWGEORGE Biotech) according to the manufacturer’s instructions.

### scRNA-seq data analysis and calculation of gene signature scores

The raw sequencing scRNA-seq data of 14 ICC samples from SC-iCCA Cohort [20] were downloaded from the Genome Sequence Archive in National Genomics Data Center under the accession number HRA000863. CellRanger (v3.1.0) was applied for processing raw fastq files. Then we used Seurat (v4) [51] to analyze the scRNA-seq data after doublets removal. The annotation of major cell types was performed according to their highly expressed marker genes. To illustrate the phenotypic differences of DCs, we used the “AddModuleScore” function implemented in Seurat to calculate the cell scores of indicated gene signatures. Phenotypic gene sets such as MHC class II protein complex (GO:0001525), antigen processing and presentation (GO:0019882), myeloid dendritic cell activation (GO:0001773), myeloid dendritic cell chemotaxis (GO:0002408) were obtained from MSigDB (https://www.gsea-msigdb.org).

### Tumor-infiltrating immune cells estimation

Based on bulk RNA-seq data of the FU-iCCA cohort [22], CIBERSORT algorithm was used to infer the proportions of 22 tumor-infiltrating immune cells in each ICC sample [21].

### Gene set enrichment analysis

Gene Set Enrichment Analysis (GSEA) [52] was used to evaluate functional enrichment of gene sets between ICC samples with LNM and those without LNM in FU-iCCA cohort.

### Immunoblot

Cultured cells were lysed in RIPA lysis buffer supplemented with protease and phosphatase inhibitors (Beyotime, China). Cell supernatants were collected after being centrifuged at 12,000 rpm for 15 min and quantified by a BCA protein concentration kit (Beyotime, China). Then, 5× SDS loading buffer was added and the samples were boiled at 100 °C for 5 min. Protein samples were separated by 10% SDS-PAGE gel and then electro-transferred onto PVDF membranes (Millipore). After blocked with 5% non-fat milk for 1 h, the membranes were incubated with primary antibodies specific for β-catenin (CST, Cat# 8480S,1:1000) and β-actin (CST, Cat# 4970S,1:1000) at 4 °C overnight, followed by HRP-conjugated secondary antibody for 2 h at room temperature. Then, membranes were washed with TBST and reactive bands were visualized using ECL substrate (Tanon).

### Tissue microarray (TMA), immunofluorescence (IF) and immunohistochemistry (IHC) staining

A tissue microarray composed of 219 paraffin-embedded ICC specimens was constructed, and IHC was performed as described previously [50, 53]. The antibodies used for IHC staining of TMAs were LYVE1 (1:2500, Cat# ab314241, Abcam), CD8 (1:100, Cat# ab101500, Abcam), CXCL12 (1:100, Cat# 17402–1-AP, Proteintech), β-catenin (1:100, Cat# 8480S, CST) and non-p-β-catenin (1:400, Cat# 8814S, CST). For staining of spontaneous murine ICCs, the antibodies were CK19 (1:1000, Cat# ab52625, Abcam), CD11c (1:400, Cat# 97585, CST), CD8 (1:2000, Cat# ab209775, Abcam). For dual-color IF staining of TMAs, the antibodies were CD11c (1:400, Cat# 45581S, CST) and CD45 (1:1000, Cat# ab40763, Abcam). Anti-CK19 (1:500, Cat# ab52625, Abcam) antibody was used for IF staining of MuCCA1 and mIC-23 cells. After IF staining, TMA slides were scanned and evaluated through Pannoramic Viewer (3DHISTECH, Budapest, HUNGARY). For IHC staining, slides were scanned and evaluated by NDP.view 2.16.13 (Hamamatsu). CXCL12 staining density was quantified by H score with the assistance of QuPath (v0.4.3) [54], an open-source software for digital pathology slide image analysis. For evaluation of tumor-infiltrating immune cells, CD8^+^ T cells and CD45^+^CD11c^+^ DCs were counted. Briefly, for each patient, three independent microscopic fields (400X) were selected and assessed by 2 investigators blinded to clinical data. The results are shown as the average value of triplicates.

### Statistical analysis

Statistical analyses were performed using R (v4.1.2) package and Graphpad 9.0 software. To compare the differences of quantitative variables between two groups, Student’s *t* test or Mann–Whitney test was used when appropriate. For single-cell analysis, comparison of two groups was performed using Wilcoxon rank sum test. The association between categorical variables was evaluated using the Chi-square test. Spearman correlation analysis was used to describe the correlation between variables. Overall survival (OS) was defined as the interval between surgery and death or the last follow-up date. Recurrence-free survival (RFS) was calculated from surgery to the date when recurrence was identified. The last follow-up date in TMA cohort was Dec 31st 2020. Kaplan–Meier survival analyses and log-rank tests were performed using the R packages survival and survminer compare the OS and RFS of two groups. The samples were divided into high and low groups according to the median value except for the survival analysis of dendritic cells in scPLC cohort, which is based on the optimal cut-point determined by R function surv_cutpoint. Two-tailed *P* < 0.05 was regarded as statistically significant.

### Supplementary information

Supplementary materials

## Data Availability

All data generated in this study are included in the article. The datasets are available from the corresponding author upon reasonable request. For public datasets, raw scRNA-seq data of ICC samples in SC-iCCA Cohort [20] are accessible at the Genome Sequence Archive in National Genomics Data Center under the accession number HRA000863.The Bulk RNA-seq data FU-iCCA cohort [22] are available in biosino NODE database (NODE database: OEP001105). Other datasets are acquired from the Gene Expression Omnibus (GEO) under the accession numbers GSE76927 and GSE107943.
