# Supplementary material for: Mobilization and activation of tumor-infiltrating dendritic cells inhibits lymph node metastasis in intrahepatic cholangiocarcinoma
Source: Cell Death Discov. 2024 Jun 26;10:304. doi: 10.1038/s41420-024-02079-z (PMC11208581; doi:10.1038/s41420-024-02079-z)

Supplementary materials

1. Supplementary Tables 2

2. Supplementary Figures 4

3. Original western blots 9

1. Supplementary Tables

Table S1. Primers for qRT-PCR

Gene names	Primer sequences (5’ to 3’)
human CTNNB1	5′-AGCTTCCAGACACGCTATCAT-3′ (F)
	5′-CGGTACAACGAGCTGTTTCTAC-3′ (R)
human CCL4	5′- CTGTGCTGATCCCAGTGAATC-3′ (F)
	5′- TCAGTTCAGTTCCAGGTCATACA-3′ (R)
human CCL5	5′- CCAGCAGTCGTCTTTGTCAC -3′(F)
	5′- CTCTGGGTTGGCACACACTT -3′ (R)
human CXCL12	5′- ATTCTCAACACTCCAAACTGTGC -3′ (F)
	5′- ACTTTAGCTTCGGGTCAATGC-3′ (R)
human β-actin	5′-CACCATTGGCAATGAGCGGTTC-3′ (F)
	5′-AGGTCTTTGCGGATGTCCACGT-3′ (R)
mouse Ctnnb1	5′-ATGGAGCCGGACAGAAAAGC-3′ (F)
	5′-CTTGCCACTCAGGGAAGGA-3′ (R)
mouse Cxcr4	5′-GAAGTGGGGTCTGGAGACTAT-3′ (F)
	5′-TTGCCGACTATGCCAGTCAAG-3′ (R)
mouse β-actin	5′-GGCTGTATTCCCCTCCATCG-3′ (F)
	5′-CCAGTTGGTAACAATGCCATGT-3′ (R)

Table S2. Primers for promoter regions of CXCL12

Location from TSS	Primer sequences (5’ to 3’)
-1200bp to -800bp	GGAGCCTGAGAAGGTCAAAG
	GGCGCTTTAGAGGGGAGA
-800bp to -400bp	ACTGCAAAGACGGGTCTCAT
	GATGGCGGGAACTGAATG
-400bp to 0bp	TCCGTGGGAAGAGTTTTCTG
	GGACGGGAGATTCAATGAGA

Table S3. Target sequences of siRNAs against CTNNB1

Gene names	Target sequences (5’ to 3’)
Human CTNNB1	Sense: 5’-GUAAUAAGCCGGCUAUUGU-3’
	Antisense: 5’-ACAAUAGCCGGCUUAUUAC-3’
Mouse Ctnnb1	Sense: 5’-GCAGAAUACAAAUGAUGUA-3’
	Antisense: 5’- UACAUCAUUUGUAUUCUGC-3’

2. Supplementary Figures

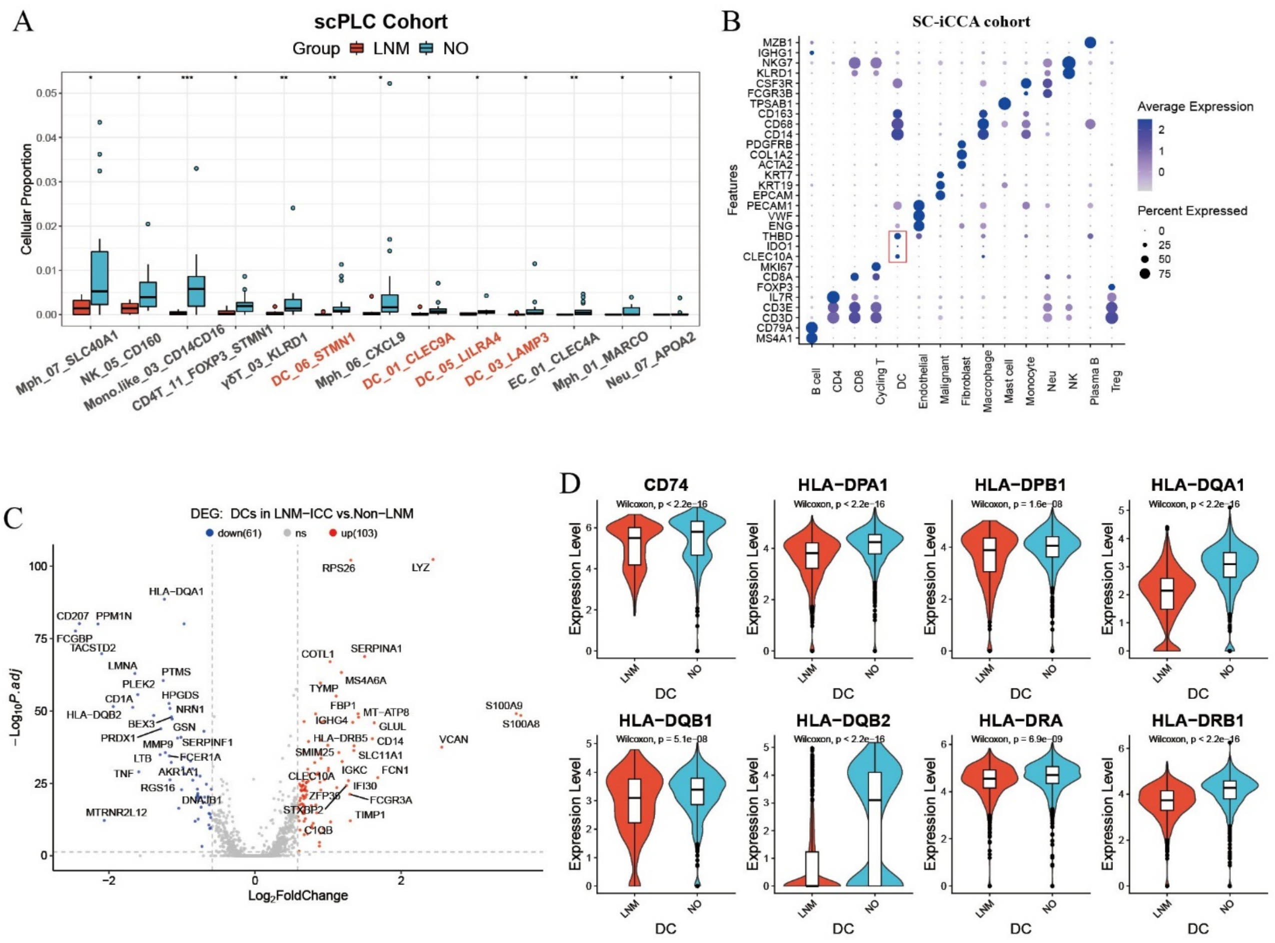

Figure S1. scRNA-seq analysis of cell type fractions between ICC samples with or without LNM. (A) Boxplots showing the fractions of differentially distributed cell subclusters identified in scPLC cohort. (B) Dot plot showing the expression pattern of marker genes of indicated cell types in SC-iCCA cohort. (C) Volcano plot showing differentially expressed genes of DCs between ICC samples with or without LNM. (D) Violin plots showing the expression of MHC-II related molecules (CD74, HLA-DP, HLA-DQ, and HLA-DR) in DCs between ICC samples with or without LNM. *p < 0.05, **P < 0.01, ***p < 0.001.

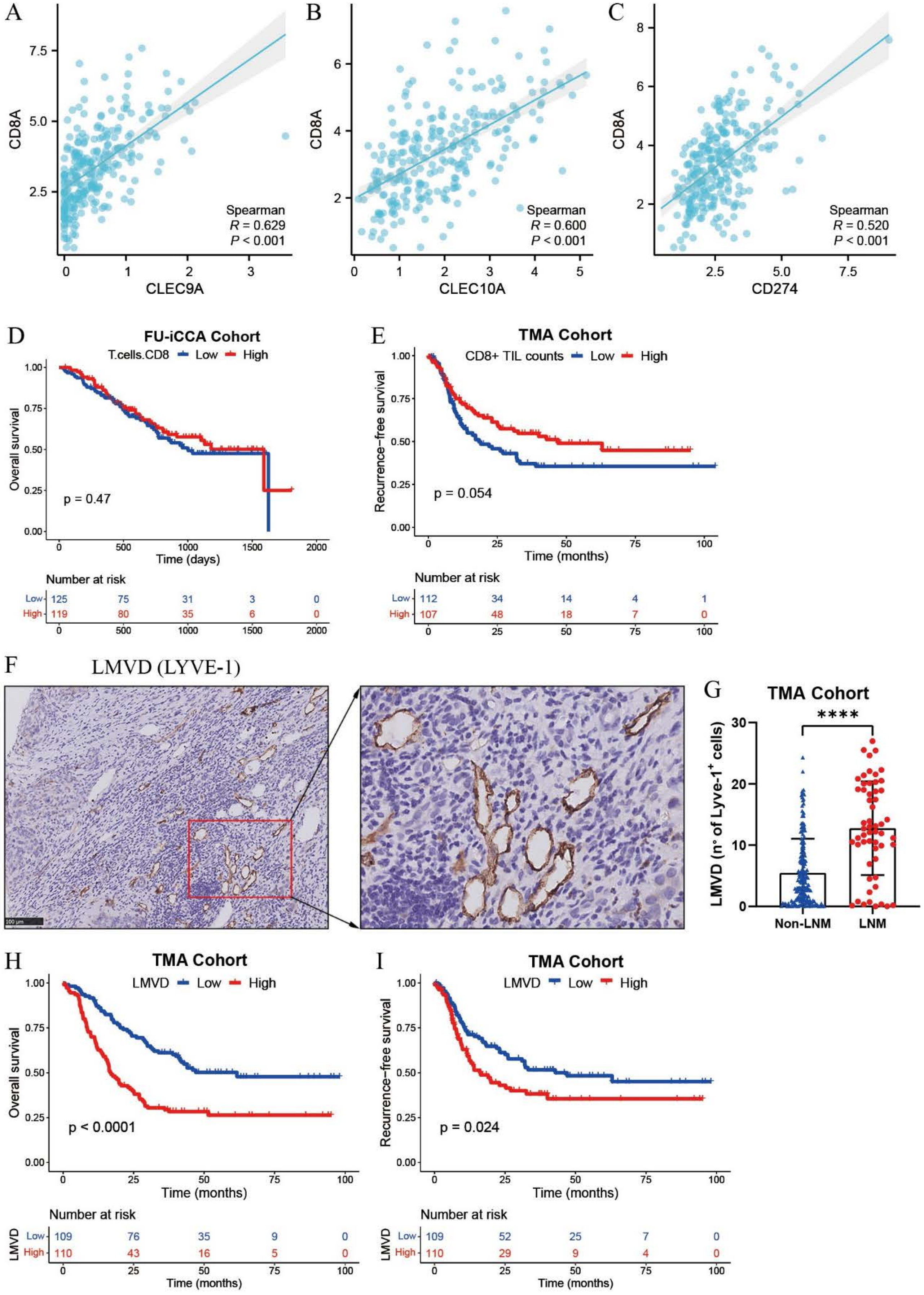

A
CD8A
CLEC9A
Spearman
R = 0.629
P < 0.001
B
CD8A
CLEC10A
Spearman
R = 0.600
P < 0.001
C
CD8A
CD274
Spearman
R = 0.520
P < 0.001
D
FU-iCCA Cohort
T.cells.CD8 Low High
Overall survival
p = 0.47
Time (days)
Number at risk
Low 125 75 31 3 0
High 119 80 35 6 0
E
TMA Cohort
CD8+ TIL counts Low High
Recurrence−free survival
p = 0.054
Time (months)
Number at risk
Low 112 34 14 4 1
High 107 48 18 7 0
F
LMVD (LYVE-1)
100 μm
G
TMA Cohort
****
LMVD (n° of Lyve-1+ cells)
Non-LNM
LNM
H
TMA Cohort
LMVD Low High
Overall survival
p < 0.0001
Time (months)
Number at risk
LMVD
Low 109 76 35 9 0
High 110 43 16 5 0
I
TMA Cohort
LMVD Low High
Recurrence−free survival
p = 0.024
Time (months)
Number at risk
LMVD
Low 109 52 25 7 0
High 110 29 9 4 0

Figure S2. Correlation of CD8A transcripts with DC marker genes and survival analysis of CD8⁺ T cells. Spearman correlation analysis of CD8A transcripts with conventional DC marker genes CLEC9A (A), CLEC10A (B) as well as PD-L1 (C) in FU-iCCA cohort. Survival analysis of ICC patients grouped by infiltration levels of tumor-infiltrating $CD8^+$ T cells in FU-iCCA cohort (D) and TMA cohort (E). Representative IHC staining images of anti-LYVE-1 (F) and quantification of LMVD (G). Kaplan-Meier survival curves for OS (H) and RFS (I) of ICC patients grouped by LMVD in TMA cohort. LMVD, lymphatic micro-vessel density. ****p < 0.0001.

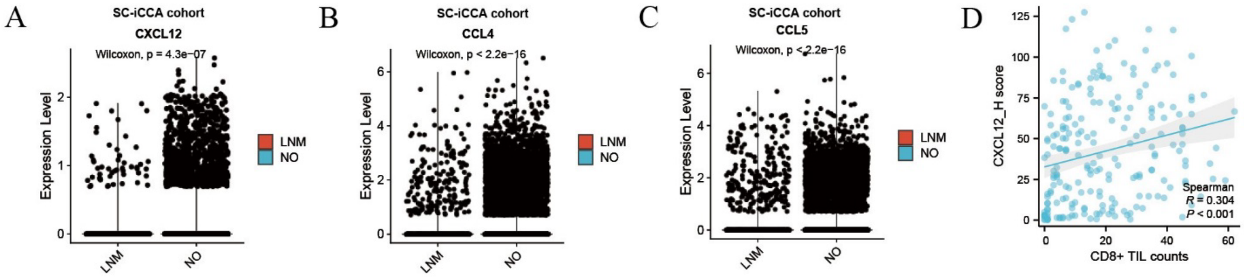

Figure S3. Defective production of chemokines in ICC malignant cells with LNM. Expression levels of chemokines CXCL12 (A), CCL4 (B), and CCL5 (C) in ICC single malignant cells with or without LNM in SC-iCCA cohort. (D) Spearman correlation of CXCL12 H-score with cell counts of $CD8^+$ TILs in TMA cohort.

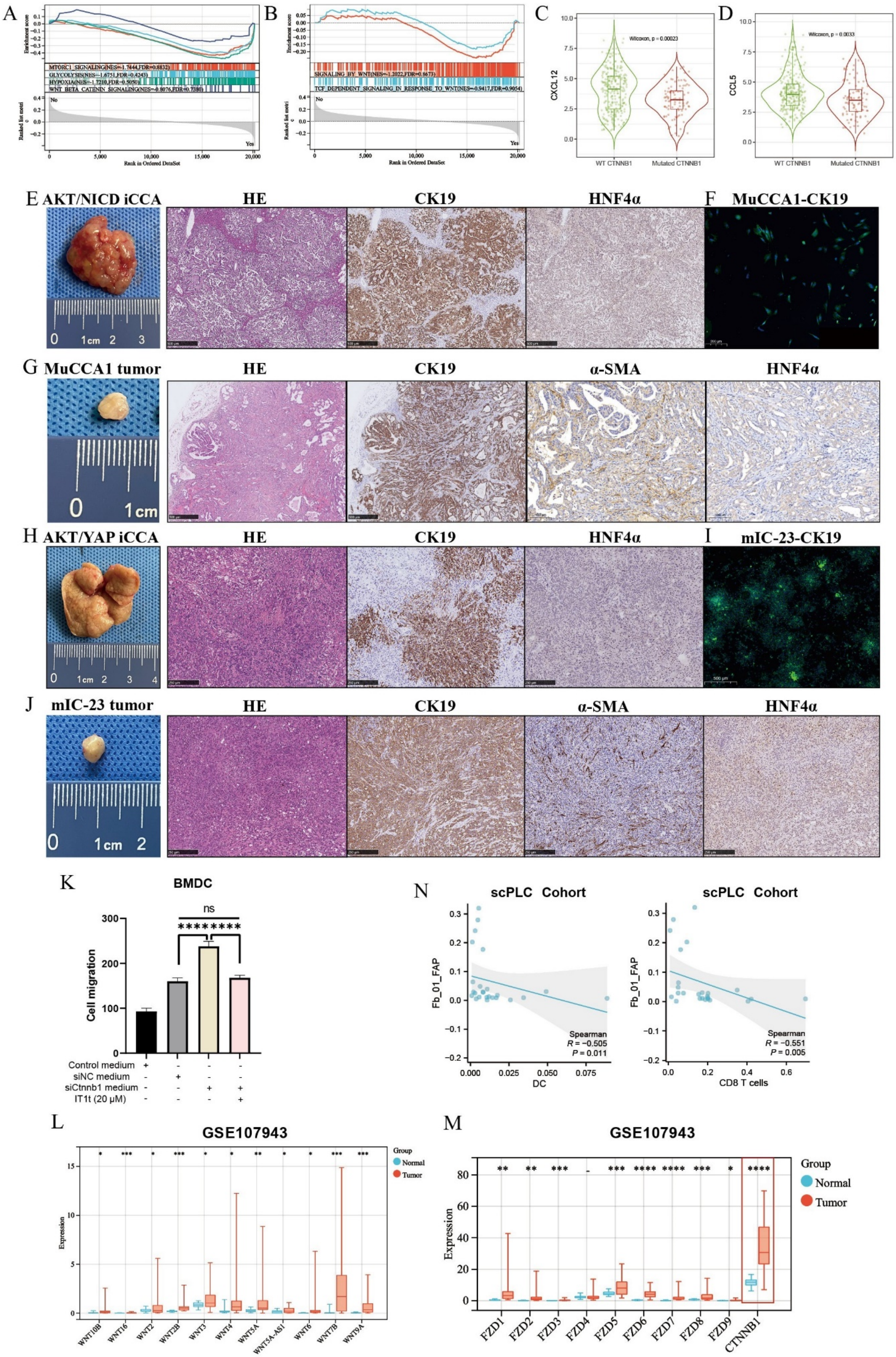

A
MTORC1_SIGNALING(NES=-1.7444,FDR=0.8832)
GLYCOLYSIS(NES=-1.6751,FDR=0.4243)
HYPOXIA(NES=-1.7210,FDR=0.5050)
WNT_BETA_CATENIN_SIGNALING(NES=-0.8076,FDR=0.7380)
Enrichment score
Ranked list metric
Rank in Ordered DataSet
B
SIGNALING_BY_WNT(NES=-1.2022,FDR=0.8673)
TCF_DEPENDENT_SIGNALING_IN_RESPONSE_TO_WNT(NES=-0.9417,FDR=0.9054)
C
Wilcoxon, p = 0.00023
CXCL12
WT CTNNB1
Mutated CTNNB1
D
Wilcoxon, p = 0.0033
CCL5
E AKT/NICD iCCA
HE
CK19
HNF4α
F MuCCA1-CK19
G MuCCA1 tumor
α-SMA
H AKT/YAP iCCA
I mIC-23-CK19
J mIC-23 tumor
K
BMDC
Cell migration
ns
Control medium
siNC medium
siCtnnb1 medium
IT1t (20 μM)
N
scPLC Cohort
Fb_01_FAP
DC
CD8 T cells
Spearman
R = −0.505
P = 0.011
R = −0.551
P = 0.005
L
GSE107943
Expression
Group
Normal
Tumor
WNT10B
WNT16
WNT2
WNT2B
WNT3
WNT4
WNT5A
WNT5A-AS1
WNT6
WNT7B
WNT9A
M
GSE107943
FZD1
FZD2
FZD3
FZD4
FZD5
FZD6
FZD7
FZD8
FZD9
CTNNB1

Figure S4. β-catenin activation impairs DC recruitment by suppressing CXCL12 expression. Gene set enrichment analysis (GSEA) of differential Hallmark (A) and REACTOME (B) gene sets based on RNA-seq data between LNM and non-LNM ICC samples in FU-iCCA cohort. Expression of CXCL12 (C) and CCL5 (D) in human CTNNB1-wild type or mutant HCC samples from TCGA-LIHC cohort. (E) Gross liver images, H&E staining and IHC staining for CK19 and HNF4α of liver sections from AKT/NICD-driven murine ICCs (scale bar: 500μm). (F) IF staining of CK19 in MuCCA1 cells (scale bar: 200μm). (G) Gross images, H&E staining and IHC staining for CK19, α-SMA and HNF4α of subcutaneous tumor tissues derived from MuCCA1 cells. (H) Gross liver images, H&E staining and IHC staining for CK19 and HNF4α of liver sections from AKT/YAP-driven murine ICCs (scale bar: 250μm). (I) IF staining of CK19 in mIC-23 cells (scale bar: 500μm). (J) Gross images, H&E staining and IHC staining for CK19, α-SMA and HNF4α of subcutaneous tumor tissues derived from mIC-23 cells (scale bar: 250μm). (K) Trans-well migration assay of BMDCs attracted by condition medium and siRNA-treated mIC-23 cell supernatants, with or without IT1t dihydrochloride (20 μM) for 24h. (L-M) Expression levels of indicated WNT ligands and FZD receptors, as well as CTNNB1 in human ICC samples compared with nontumorous surrounding liver tissues based on the RNA-seq dataset GSE107943. (N) Spearman correlation analysis between Fb_01_FAP and the infiltration of DC and CD8 T cells in scPLC Cohort.

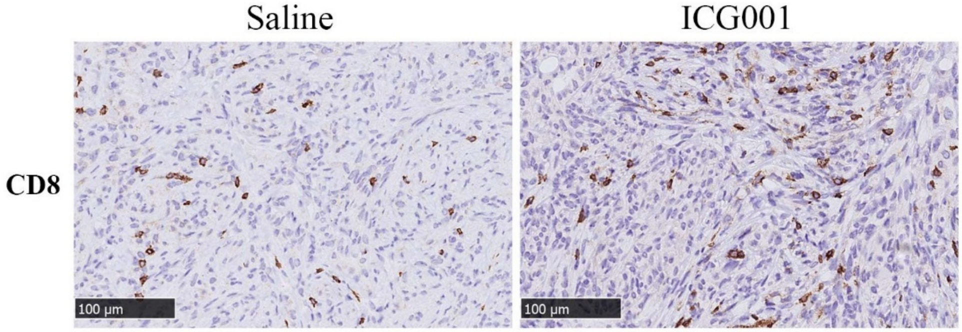

Figure S5. β-catenin inhibitor ICG-001 promoted intra-tumoral CD8$^+$ T cell infiltration. Representative IHC staining images of CD8$^+$ T cells in mIC-23 footpad tumors from indicated groups.

3. Original western blots

Figure 4F

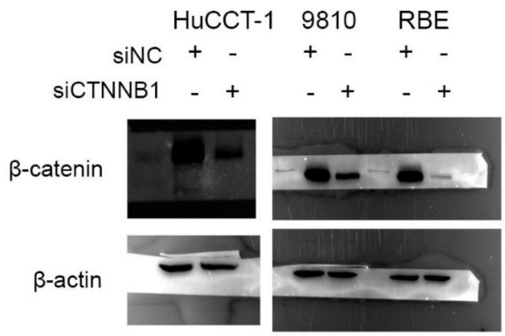

Figure 4L

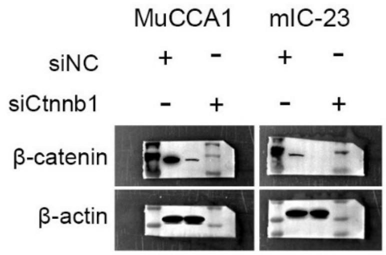

Supplement: Supplementary file 1 — Supplementary materials [file 41420_2024_2079_MOESM1_ESM.pdf]
